# HIF-1α/YAP Signaling Rewrites Glucose/Iodine Metabolism Program to Promote Papillary Thyroid Cancer Progression

**DOI:** 10.7150/ijbs.75459

**Published:** 2023-01-01

**Authors:** Hongjun Song, Zhongling Qiu, Yang Wang, Chuang Xi, Guoqiang Zhang, Zhenkui Sun, Quanyong Luo, Chentian Shen

**Affiliations:** Department of Nuclear Medicine, Shanghai Sixth People's Hospital Affiliated to Shanghai Jiao Tong University School of Medicine. Shanghai, China.

**Keywords:** Papillary thyroid cancer, Glucose/Iodine metabolism, HIF-1α, YAP, Progression.

## Abstract

**Background:** The management of aggressive and progressive metastatic papillary thyroid cancer (PTC) is very difficult. An inverse relationship between radioiodine and F-18 fluorodeoxyglucose (FDG) uptake (''flip-flop'' phenomenon) is described for invasive PTC during dedifferentiation. However, no satisfactory biologic explanation for this phenomenon. Hypoxia is an important microenvironmental factor that promotes cancer progression and glycolysis. The Hippo-YAP is a highly conserved tumor suppressor pathway and contributes to cancer metabolic reprogramming. Thus, we investigated the underlying molecular mechanisms of glucose/iodine metabolic reprogramming in PTC, focusing on the tumor hypoxia microenvironment and Hippo-YAP signaling.

**Methods:** Immunohistochemistry staining was conducted to evaluate the expressions of hypoxia-inducible factor 1α (HIF-1α), yes-associated protein (YAP), glucose transporters 1 (GLUT1) and sodium iodine symporter (NIS) in matched PTC and the adjacent noncancerous tissues. PTC cell lines were cultured under normoxic (20% O_2_) and hypoxic (1% O_2_) conditions and the glycolysis level and NIS expression were measured. Further, we characterized the molecular mechanism of glucose/iodine metabolic reprogramming in PTC cell. Finally, we validated the results in vivo by establishing subcutaneous xenografts in nude mice.

**Results:** The expression levels of HIF1-α, YAP and GLUT1 were upregulated in PTC tissues and YAP expression was positively associated with HIF-1α, GLUT1 and TNM stages. Meanwhile, the expression of NIS was negatively correlated with YAP. Further, in vitro studies indicated that hypoxia-induced YAP activation was critical for accelerating glycolysis and reducing NIS expression in PTC cells. Inhibition of YAP had the opposite effects in vitro and tumorigenicity in vivo. Hypoxia inhibited the Hippo signaling pathway resulting in the inactivation of YAP phosphorylation, further promoting the nuclear localization of YAP in PTC cells. The mechanism is that hypoxic stress promoted YAP binding to HIF-1α in the nucleus and maintained HIF-1α protein stability. The YAP/HIF-1α complex bound and directly activated the GLUT1 transcription to accelerate glycolysis. Meanwhile, HIF-1α/YAP signaling might indirectly reduce the expression of NIS by promoting the output of MAPK signaling. In vivo studies confirmed the YAP-mediated reprogramming of glucose/iodine metabolism promoted PTC progression.

**Conclusions:** Collectively, our data revealed a novel regulatory mechanism of the glucose/iodine metabolic program rewritten by HIF-1α/YAP signaling in PTC. Inhibition of HIF-1α/YAP signaling alone or in combination with other potential markers may effectively combat aggressive PTC.

## Introduction

Papillary thyroid cancer (PTC) usually has a favorable prognosis. However, the management of aggressive and advanced metastatic PTC, especially radioactive iodine-refractory (RAIR), is challenging for the clinicians 1. The RAIR-PTC, usually a sign of higher aggressiveness, less differentiated and worse prognosis, can be detected by [F-18]-fluorodeoxyglucose positron emission tomography/computed tomography (^18^F-FDG-PET/ CT) 2. In particular, ^18^F-FDG-PET/ CT is considered useful for the detection of recurrent or metastatic thyroid cancer in patients with negative radioiodine scans, especially those with a high thyroglobulin (Tg) level. There is an inverse relationship between iodine and FDG accumulation, the so-called ''flip-flop'' phenomenon, that is when differentiated thyroid cancer cells dedifferentiate they tend to lose their affinity for iodine with increased glucose demand 3. Thus, it is commonly accepted that high ^18^F-FDG uptake reflects poor differentiation in thyroid cancers. However, the molecular mechanisms underlying glucose/iodine metabolic reprograming in dedifferentiated thyroid cancer are not entirely comprehended.

The ''flip-flop'' phenomenon was thought to be a consequence of reduced or lost sodium iodide symporter (NIS) expression and increased glucose transporter-1 (GLUT1) 3. The expression of NIS was vital for the iodide accumulation and formed the basis for the treatment of PTC with radioactive iodine. In thyroid cancer, downregulation of NIS expression was proportional to the extent of dedifferentiation and was well studied. 4. Recently, the relationship between tumor differentiation and glucose metabolism in thyroid cancer was investigated. It was considered that thyroid malignant cells become more eager for glucose during dedifferentiation 5. Hence, tumor cells rearrange their metabolism to meet their excess glucose demands by upregulation of GLUT1, to promote cell growth and survival 6.

Overexpression of GLUT1 has been shown to be an indicator of greater biological aggressiveness and loss of tumor differentiation in thyroid cancer 5. The deregulation of glucose metabolism in tumor cells is predominantly mediated by oxygen-related transcription factors, such as the hypoxia-inducible factor 1α (HIF-1α). HIF-1α is found higher in the most aggressive dedifferentiated thyroid cancers 7. Hypoxia microenvironment is a common feature of various malignant tumors and contributes to cancer progression and more aggressive phenotype selection. To adapt to hypoxic stress, tumor cells are capable of altering their glucose metabolism from oxidation to glycolysis, which is called Warburg effect 8. HIF-1α is suggested to contribute to the Warburg effect by stimulating many genes that mediate glycolysis, including GLUT1, which possess hypoxia-response elements (HREs) in its promoter 9.

Metabolic reprogramming is not only regulated by factors such as HIF-1α, but also by dysregulation of certain signaling pathways that also contribute to cancer metabolic remodeling 10. Among them, the Hippo signaling pathway is a highly conserved tumor suppressor pathway, and yes-associated protein (YAP) is one of the two effectors of the pathway 11. The phosphorylation status and localization of YAP determines its activity; and YAP activation via nuclear localization is the most important regulatory mechanism 12. Extensive research has been performed on YAP regarding its oncogenic potential, such as modulation of tumor invasive behavior. Furthermore, YAP is frequently overexpressed in PTC, and its activation is associated with the extrathyroidal extension and distant metastasis 13-15. RAS genes, which are associated with poorly PTC, are themselves transcriptionally regulated by YAP 16. Induction of RAS signaling by inactivation of Hippo results in aberrant activation of mitogen-activated protein kinase (MAPK) output, which is the most important pathway contributing to the occurrence, development and reduction of NIS in PTC 17. Interestingly, the role of YAP is not limited to its oncogenic potential, but has also been shown to affect glucose metabolism by upregulation of genes involved in glycolysis and glucose transporters, such as GLUT1, which is also regulated by HIF-1α 11, 18. Furthermore, studies have found that YAP interacted with HIF-1α to directly activates the transcription of glucose metabolism genes and accelerate glycolysis in cancer cells 19-20. Therefore, it would be vital to determine the potential regulatory mechanism of YAP and HIF-1α in reprograming glucose/iodine metabolism in PTC.

## Materials and Methods

### Patients and Immunohistochemistry

This study was approved by the Clinical Research Ethics Committee of Shanghai Jiao Tong University Affiliated Sixth People's Hospital (Shanghai, China), and written informed consent was obtained from each patient. Between 2014 and 2017, we obtained 108 tumor tissues and the paired adjacent noncancerous tissues from PTC patients who had undergone thyroidectomy at our hospital. Surgical specimens were formalin-fixed and paraffin-embedded to establish tissue microarrays (TMA) for subsequent immunohistochemical (IHC) staining analysis. In January 2020, another 10 pairs (cancer and adjacent noncancer tissues) of fresh specimens from PTC patients were obtained. These thyroid tissue samples were processed immediately after sectioning and were stored in liquid nitrogen for subsequent Real-time PCR and western blotting. The inclusion criteria of patients were as follows (a) histopathological diagnosis of PTC and no history of other malignancies; (b) no other preoperative treatment; (c) complete medical history. TNM stages were collected from each patient and classified according to the American Joint Committee on Cancer (AJCC, 8th edition) criteria for differentiated thyroid cancer (DTC). PTC specimens TMA were used for IHC staining analysis. Primary antibodies against HIF-1α (1:50), YAP (1:100), GLUT1 (1:100) and NIS (1:50) were used. The immunohistochemical procedure was performed as previously described 21. The staining intensity and extent were scored independently by two pathologists blinded to the patient information, using the semiquantitative immunoreactivity scoring system as previously described 7. The staining intensity and extent were multiplied to generate immunohistochemistry scores.

### Cell Culture and Regents

The BCPAP and TPC-1 cell lines were purchased from the Institute of Biochemistry and Cell Biology (SIBS, CAS, Shanghai, China). All cell lines have been confirmed to contain the BRAF^V600E^ mutation, short tandem repeat, and amelogenin authentication as described in the ANSI Standard by the ATCC Standards Development Organization (SDO). All cell lines were maintained in RPMI 1640 medium (Cat. no.11875-093; Gibco) supplemented with 10% fetal bovine serum (FBS, Cat. no. 16000-044; Gibco) in a 5% CO_2_ atmosphere at 37°C. PTC cells were cultured under normoxic (20% O_2_) or hypoxic conditions (1% O_2_). In the study, all compounds were obtained from Selleck and used at the indicated concentrations.

### Western Blotting, Transwell migration and invasion assays

Western blotting, migration and invasion assays (transwell and wound healing assays) were conducted as previously described 22, 23, 24. Supplementary Table 1. shown the information about the antibodies used in the study.

### RNA extraction, real-time PCR and RNA interference

RNA were extracted with TRIzol (Invitrogen, Carlsbad, CA, USA) and real-time PCR were performed as described previously 23. The primers used are listed in **Supplementary file 1**. PTC cell transfections were performed using Invitrogen Lipofectamine 3000 (Thermo Fisher Scientific, Shanghai, China) as per instructions from the manufacturer. After 48h later, PTC cells were harvested to perform real-time PCR or western blotting. The sense sequences of the YAP small interfering RNAs (siRNAs) (siYAPs, Sigma, Shanghai, China) were as follows:

YAP siRNA: GTAGCCAGTTACCAACACT

### Extracellular acidification rate (ECAR) and glucose level measurement

In the study, we used Seahorse XF24 Extracellular Flux Assay Kits (Agilent Technologies) for determination of the extracellular acidification rate (ECAR). According to the manufacturer's protocols, ECAR was examined with a Seahorse XF glycolysis stress test kit. BCPAP and TPC-1 cells were seeded overnight into a Seahorse XF24 cell culture plate at a density 1 × 10^4^ cells/well. After baseline measurements, Seahorse injection ports were loaded with glucose, oligomycin, and 2-DG at the indicated time points. ECAR data were evaluated by Seahorse XF-96 Wave software. To measure glucose uptake, PTC cells were incubated with 100 µg/ml 2-deoxy-2-[(7- nitro-2,1,3-benzoxadiazol-4-yl)amino]-D-glucose (2-NBDG, Abcam, #ab235976) in glucose-free medium for one hour and the fluorescence was measured at excitation and emission wave lengths of 485nm and 535nm, respectively.

### Subcellular fractionation

PTC tissues and adjacent noncancer tissues cytoplasmic and nuclear extracts were separated using a Nuclear and Cytoplasmic Protein Extraction Kit (Beyotime, Shanghai, China) according to the manufacturer's instructions. HIF-1α, YAP and GLUT1 expressions were analyzed by western blotting.

### Immunofluorescence Assay

The PTC cell lines were cultured, washed with phosphate-buffered saline (PBS), and fixed in 4% polyformaldehyde. Primary antibody against HIF-1α (1:100) or YAP (1:100) was added on the cell plates. After being blocked with 5% goat serum for 2h, the plates were incubated overnight at 4°C. Then cells were washed and incubated with fluorescein isothiocyanate (FITC)-labeled (1:100) secondary antibody (Beyotime, Shanghai, China) for 2h. We counterstained the cells using diaminophenylindole (DAPI, Beyotime, Shanghai, China) and captured confocal images using a confocal microscope.

### Dual-luciferase reporter assay

The GLUT1 promoter reporter plasmid was amplified and inserted into the pGL3-promoter vector (Promega, Madison). PTC cells were co-transfected with GLUT1 promoter reporter plasmid (wild type or mutant) and luciferase plasmid using Invitrogen Lipofectamine 3000 (Thermo Fisher Scientific, Shanghai, China) according to the instructions from manufacturer. Cells were then cultured for 24 hours under hypoxic conditions (1% O2) or nomoxic condition (20% O2). Luciferase activity was analyzed using a dual-luciferase reporter assay (Promega) according to the manufacturer's instructions.

### Chromatin immunoprecipitation (ChIP) assay

The PTC cells were divided into control, YAP siRNA transfected and CA3-treated group. The cells were cultured under hypoxic (1% O_2_) or normoxia (20% O_2_) conditions for 24h. Next, a ChIP assay was proceeded using the EZ‐ChIP kit (Millipore) according to the manufacturer's instructions. Briefly, formaldehyde cross‐linked chromatin was sonicated to generate chromatin fragments (200 to 300 bp). Then, the fragments were immunoprecipitated with antibodies against YAP, HIF‐1α, or IgG. Finally, immunoprecipitated DNAs were analyzed by qRT‐PCR. Primer information used in ChIP analysis is as follows:

GLUT1 Forward: 5′-TCTGGCATCAACGCTGTCTTC-3′

Reverse: 5′-CGATACCGGAGCCAATGGT-3′

### Co-immunoprecipitation

After culturing under hypoxia (1% O2) for 24 hours, PTC cells were harvested and incubated with lysis buffer with protease inhibitors for 40 min on ice. Next, the supernatant was collected. HIF-1α, YAP, or immunoglobulin G (IgG) antibody was added and incubated at 4 °C overnight. Protein A/G-agarose beads was added and shaken at 4°C. The pelleted cells were collected and washed with lysis buffer. Finally, the precipitate was boiled with loading buffer for 5 min and analyzed by western blotting.

### Cycloheximide (CHX) chase and Ubiquitination assay

To determine the half-life of HIF-1α, CHX chase assay was used. PTC cells of the control and YAP siRNA group were seeded and cultured under hypoxia (1% O_2_) for 24h and treated with CHX (100 μg/ml) for 0h, 2h and 4h. The PTC cells were harvested at the specified time points and analyzed for HIF-1α expression by western blotting. For ubiquitination assay, PTC cells (the control and YAP siRNA group) were transiently transfected with ub-Flag for 24h and then incubated with 10 μM MG132 for 6h under either hypoxia (1%O_2_) or normoxia (20%O_2_). Then, the PTC cells were harvested and lysed in the denature lysis buffer. The ubiquitinated HIF1-α protein was purified and immunoblotted with anti-Flag antibodies.

### Establishment of Subcutaneous Xenografts in Nude Mice

All protocols involving mice were evaluated and approved by our Institutional Animal Care and Use Committee and performed under veterinary supervision. BALB/c mice (4-5 weeks old, 18-22 g, male) were injected subcutaneously with 5x10^6^ normal TPC-1 cells in the right flank. After 2 weeks, the inoculated mice were randomly divided into 2 groups and administered with CA3 (subcutaneous injection, 1 mg/kg/d) or control. On the 30th day, the mice were sacrificed by cervical dislocation, and primary tumors were excised and weighed. Tumor volumes were calculated and mouse body weight were measured every two day.

### Statistical analysis

Data are presented as the mean±standard deviations of three independent experiments. Graphpad Prism (version 6.01; GraphPad Software, Inc., CA) was used for the statistical analyses. The Student's t-test was used to measure comparisons between groups. A correlation coefficient was analyzed by the Pearson test. Differences with a value of p <0.05 were considered statistically significant.

## Results

### HIF1-α, YAP and GLUT1 were upregulated in PTC tissues, and NIS was down-regulated. YAP expression was positively associated with HIF-1α, GLUT1 and TNM stages, and negatively correlated with NIS

**Figure 1:**
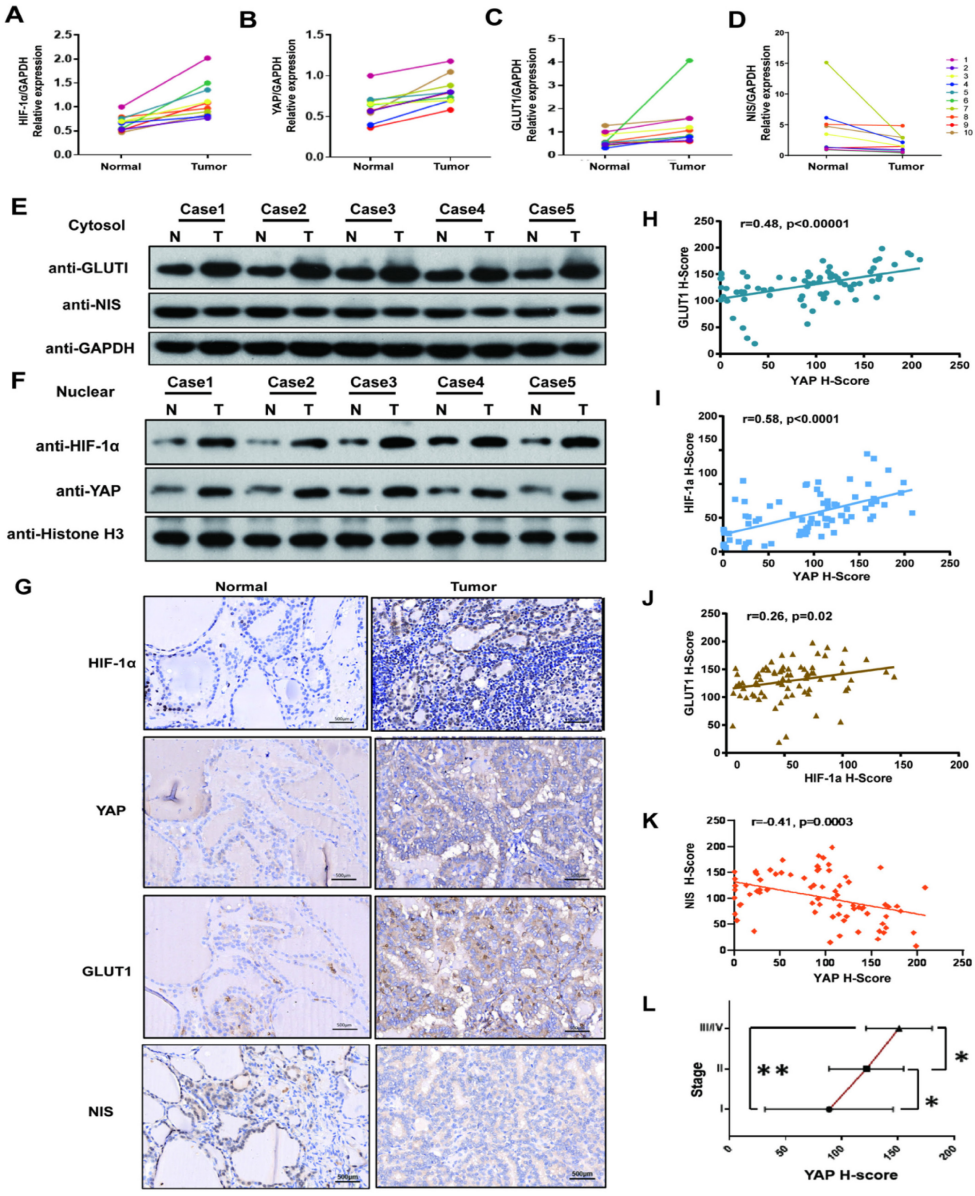
YAP expression was high in PTC tissues and correlated strongly with HIF-1α, GLUT1, NIS and TNM stages. **(A-C)** The mRNAs of HIF-1a, YAP and Glut1 are relatively overexpressed in 10 pairs of PTC tissues compared with adjacent normal tissues by real-time PCR. (**D**) The mRNA of NIS is relatively low levels of expression in 10 pairs of PTC tissues compared with adjacent normal tissues by real-time PCR. (E) Western blot showed the representative expression of GLUT1 and NIS in the cytoplasm in 5 pairs of PTC tissues and adjacent tissues. (F) Western blot showed the representative expressions of HIF-1a and YAP in the nuclear fraction in 5 pairs of PTC tissues and adjacent tissues. (G) HIF-1a, YAP and GLUT1 are overexpressed in PTC tissues, while the expression of NIS is reverse, compared with paired adjacent normal tissues by IHC (each staining brown, magnification, ×400). (**H**) IHC showed YAP correlated strongly with GLUT1 in 108 PTC tissues (p < 0.00001). (**I**) YAP and HIF-1a are positively correlated with a p < 0.0001. (**J**) The results showed a positive correlation between HIF-1a and GLUT1 (p=0.02). (**K**) The results showed a negative correlation between YAP and NIS (p=0.0003). (**L**) YAP expression is significant positively associated with TNM stages in PTC (Student's t-test, *p < 0.05, **p < 0.0001).

We firstly investigated the HIF1-α, YAP, GLUT1 and NIS expressions in 10 pairs of PTC tissues and adjacent nontumor tissues by real-time PCR. The results showed the mRNA levels of HIF1-α, YAP and GLUT1 in the PTC specimens were significantly higher than those in paracancerous tissues, while the expression of NIS was opposite. (p<0.05, Fig. 1A-D). Then cell fractionation assays were used to investigate HIF1-α, YAP and GLUT1 localizations. In PTC tissues, GLUT1 showed stronger staining in the cytoplasm, whereas YAP and HIF1-α showed stronger nuclear staining compared with paracancerous tissues (Fig. 1E-F). In addition, we further expanded the sample size and verified the relationship among HIF1-α, YAP, GLUT1 and NIS in 108 paired PTC tissues and paracancerous tissues by using imunohistochemistry. Representative staining examples are shown in Figure 1G. The expression levels of HIF1-α, YAP and GLUT1 in the PTC specimens were significantly higher than those in paracancerous tissues, while NIS expression was decreased in PTC tissue. Additionally, the expression levels of HIF1-α, YAP and GLUT1 were significantly positively correlated with each other in 108 PTC tissues (Fig. 1H-J, YAP vs GLUT1, r=0.48, p<0.00001; YAP vs HIF1-α, r=0.58, p<0.0001; HIF1-α vs GLUT1, r=0.26, p=0.02). Meanwhile, NIS was negatively correlated with YAP (Fig. 1K, YAP vs NIS, r=-0.41, p=0.0003). What's more, YAP expression was positively correlated with TNM stages (Fig. 1J). Therefore, the results demonstrate that HIF1-α, YAP and GLUT1 were upregulated in PTC tissues, but NIS was downregulated. Both HIF1-α and YAP are localized to the nucleus. Furthermore, YAP expression was positively associated with HIF-1α, GLUT1 and TNM stages and negatively correlated with NIS.

### Hypoxia promoted PTC cell glycolysis, migration and invasion in vitro

**Figure 2:**
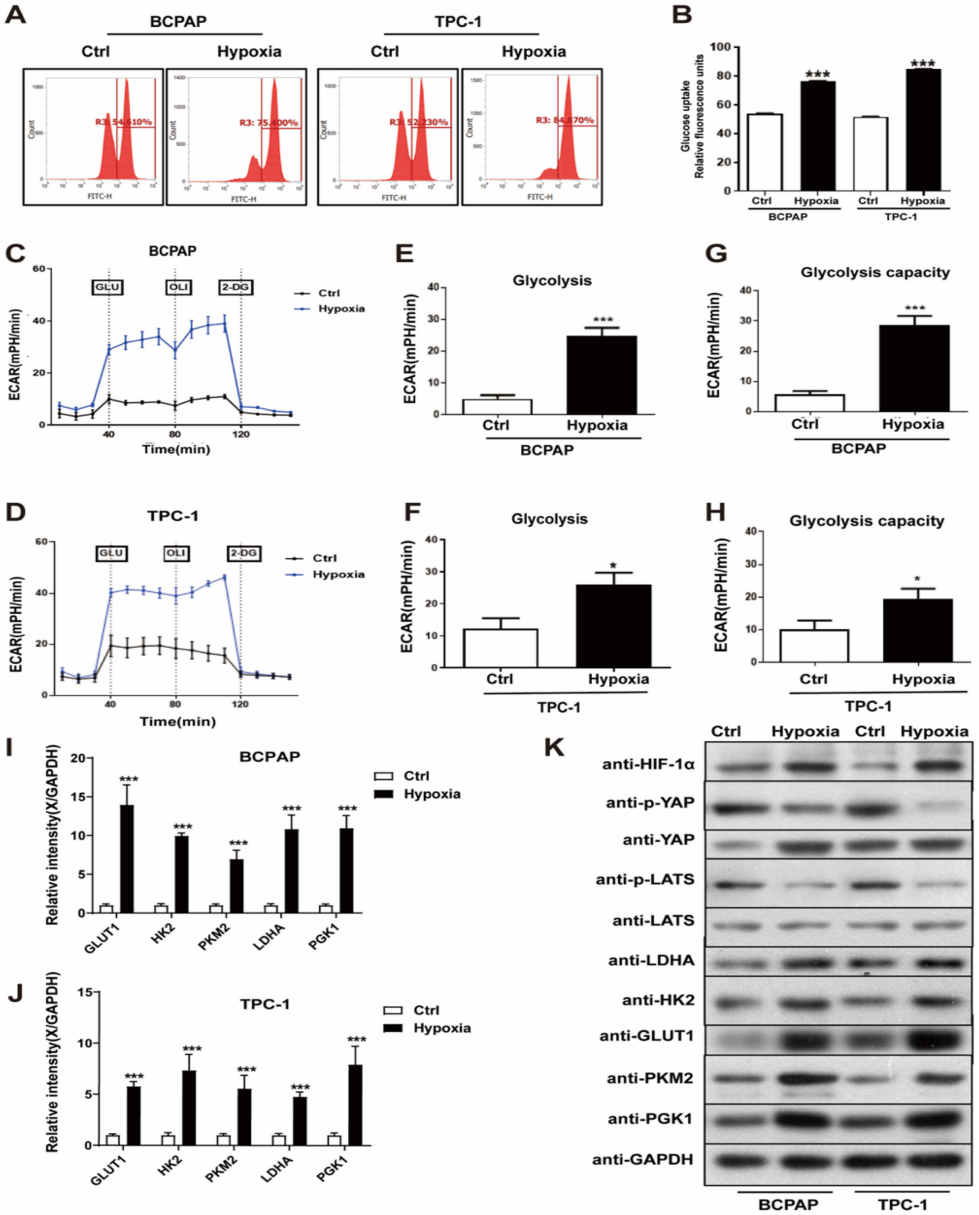
Hypoxia promoted PTC cell glycolysis in vitro. (**A-B**) Relative glucose uptake in BCPAP and TPC-1 cells under normoxia (Ctrl, 20% O_2_) or hypoxia (1% O_2_) for 24h. (**C-D**) Glycolysis reflected by extracellular acidification rate (ECAR) was measured in BCPAP and TPC-1 cells following the addition of glucose (GLU, 10 mM), oligomycin (OLI, 1 µM), and 2-deoxyglucose (2-DG, 50 mM) under normoxia (20%O_2_) or hypoxia (1%O_2_). (**E-F**) Glycolysis after the addition of glucose in BCPAP and TPC-1 cells. (**G-H**) Glycolysis capacity after the addition of oligomycin in BCPAP and TPC-1 cells. (**I-J**) Real-time PCR analysis for the expression of GLUT1, HK2, PKM2, LDHA and PGK1 genes in BCPAP and TPC-1 cells under normoxia (20%O_2_) or hypoxia (1%O_2_) for 24h, respectively. (**H**) Western blot showed the expression of HIF-1a, YAP, LATS, LDHA, HK2, GLUT1, PKM2, and PGK1 proteins in BCPAP and TPC-1 cells under normoxia (20%O_2_) or hypoxia (1%O_2_) for 24h, respectively.

**Figure 3:**
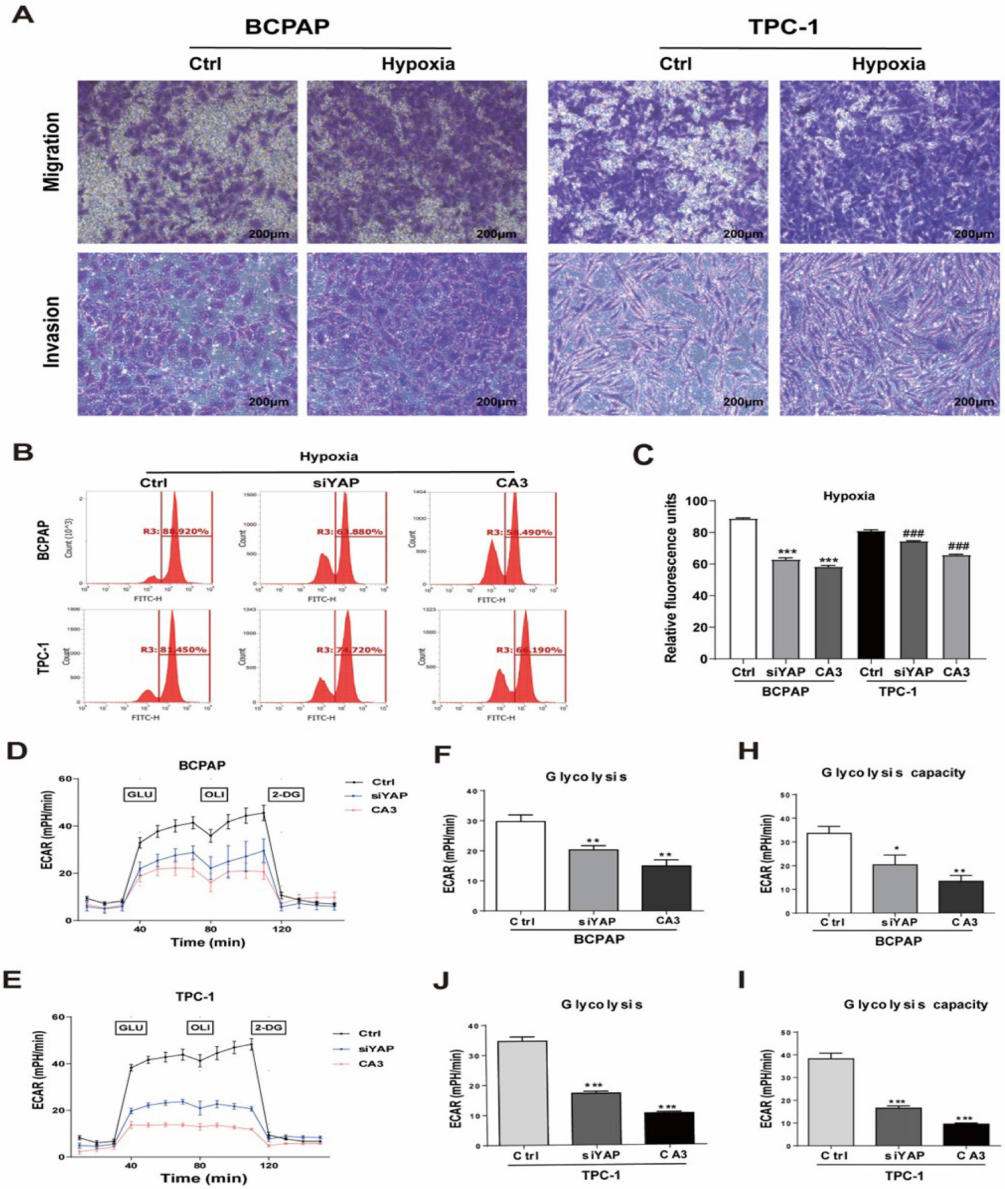
Genetic (silencing YAP) or pharmacologic (CA3) inhibition of YAP inhibited PTC cell glycolysis under hypoxia. (**A**) Migration and invasion assays in BCPAP and TPC-1 cells under normoxia (20% O_2_) or hypoxia (1% O_2_) for 24h. (magnification, × 100). Data are shown as the mean ± SEM of three independent experiments. *p < 0.05, **p < 0.01, ***p < 0.001. (**B-C**) Analysis of the uptake of glucose in BCPAP and TPC-1 cells under hypoxia (1%O_2_) for 24h after transfected with YAP siRNA or the use of the inhibitor CA3. (D**-E**) The overall glycolytic flux of PTC cells under hypoxia (1%O_2_) after transfected with YAP siRNA or the use of the inhibitor CA3 were analyzed by ECAR using seahorse instrument. (**F-J**) Glycolysis after the addition of glucose in BCPAP and TPC-1 cells. (**H-I**) Glycolysis capacity after the addition of oligomycin in BCPAP and TPC-1 cells.

To characterize the potential effects of hypoxia on PTC cell glycolysis, migration and invasion, which are required for tumorigenesis and metastasis, we performed a series of studies to evaluate them in vitro. PTC cell lines, BCPAP and TPC-1, were divided into the control (20% O_2_) and hypoxia (1% O_2_) group. The glucose assay was performed and the result showed that hypoxia significantly increased PTC cells glucose uptake, compared with normoxia (Fig. 2A and B). Hypoxia also promoted an increase extracellular acidification rate (ECAR) in PTC cells, which reflected overall glycolytic flux (Fig. 2C-H). Generally, hypoxia promotes glycolysis in cancer cells by upregulating the expression of key glycolysis enzymes. Thus, to detect the expression of key glycolysis enzymes in PTC cells, we performed real-time PCR and western blotting. The mRNAs of GLUT1, HK2, PKM2, LDHA and PGK1 expression levels were upregulated in the hypoxic cells than in the normoxic (Fig. 2I-J), western blotting yielded similar results (Fig. 2K). Besides, we examined PTC cell invasion and migration under normoxia and hypoxia using Transwell assays. Figure 3A shown that hypoxia significantly promoted PTC cell migration and invasion compared with control (Supplementary Fig. 1A and B). These results indicated that hypoxia promotes glycolysis, migration and invasion of PTC cells.

### Silencing YAP or CA3 treatment inhibited PTC cell glycolysis under hypoxia stress

**Figure 4:**
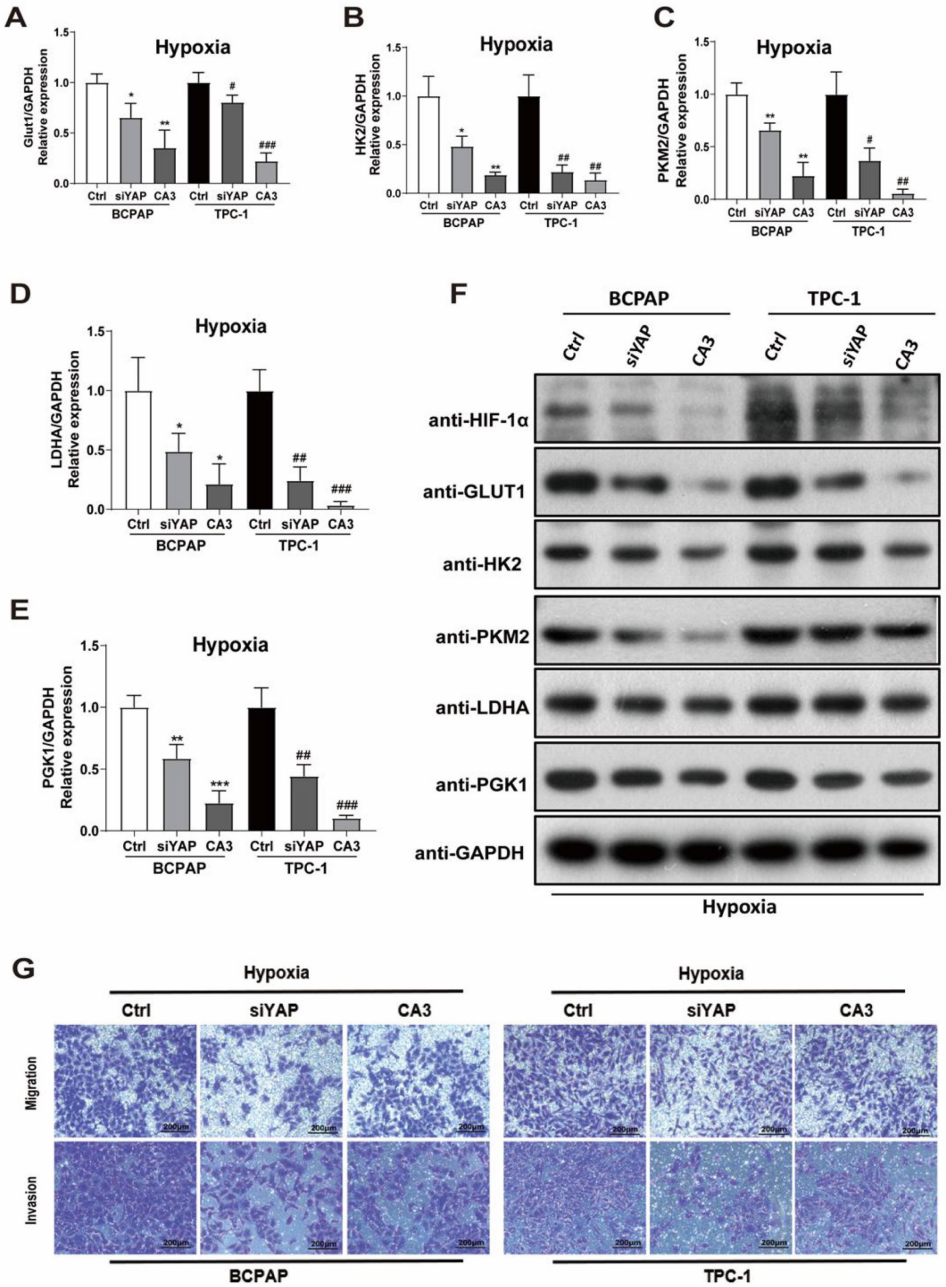
Inhibition of YAP attenuates PTC cell glycolysis, migration, and invasion under hypoxia. (**A-E**) Real-time PCR showed the mRNA expression of GLUT1, HK2, PKM2, LDHA and PGK1 genes in PTC cells under hypoxia (1%O_2_) for 24h with the inhibition of YAP. (**F**) Western blot showed the expression of HIF-1a, GLUT1, HK2, PKM2, LDHA and PGK1 proteins in BCPAP and TPC-1 cells under hypoxia (1%O_2_) for 24h with the inhibition of YAP. Data are shown as the mean ± SEM of three independent experiments. (**G**) Migration and invasion assays in BCPAP and TPC-1 cells under hypoxia (1% O_2_) for 24h after transfected with YAP siRNA or the use of the inhibitor CA3. (magnification, × 100). *p < 0.05, **p < 0.01, ***p < 0.001, ^#^p < 0.05, ^##^p < 0.01, ^###^p < 0.001.

To investigate the role of YAP in hypoxic PTC cells, two methods of inhibiting YAP were used, gene knockdown (YAP siRNA) and CA3 treatment. The efficiency of YAP siRNA knockdown was examined using real-time PCR and western blotting (Supplementary Fig. 2I-L). CA3 is a potent inhibitor of YAP transcriptional activity. As shown in Figure 3, treatment with YAP siRNA and CA3 under hypoxia both significantly decreased the PTC cell glucose uptake (Fig. 3B-C) and extracellular acidification rate (Fig. 3D-I) compared with control. These results indicated chemical or genetic inhibition of YAP in PTC cells might decreased cell glycolysis under hypoxia. To further confirm the results, we performed real-time PCR and western blotting to detect the expression of key glycolysis enzymes in PTC cells under hypoxia. The mRNAs of GLUT1, HK2, PKM2, LDHA and PGK1 expression levels were decreased in PTC cells (Fig. 4A-E) and western blotting further confirmed the results (Fig. 4F). In addition, inhibiting YAP significantly reduced the migration and invasion of hypoxic PTC cells (Fig. 4G, Supplementary Fig. 1C and D). These data suggested that YAP played a crucial role in hypoxia-induced glycolysis, migration and invasion in PTC cells.

### Hypoxia activated YAP and induced YAP nuclear translocation; HIF1-α and YAP bound and activated the GLUT1 promoter, enhancing its expression

**Figure 5:**
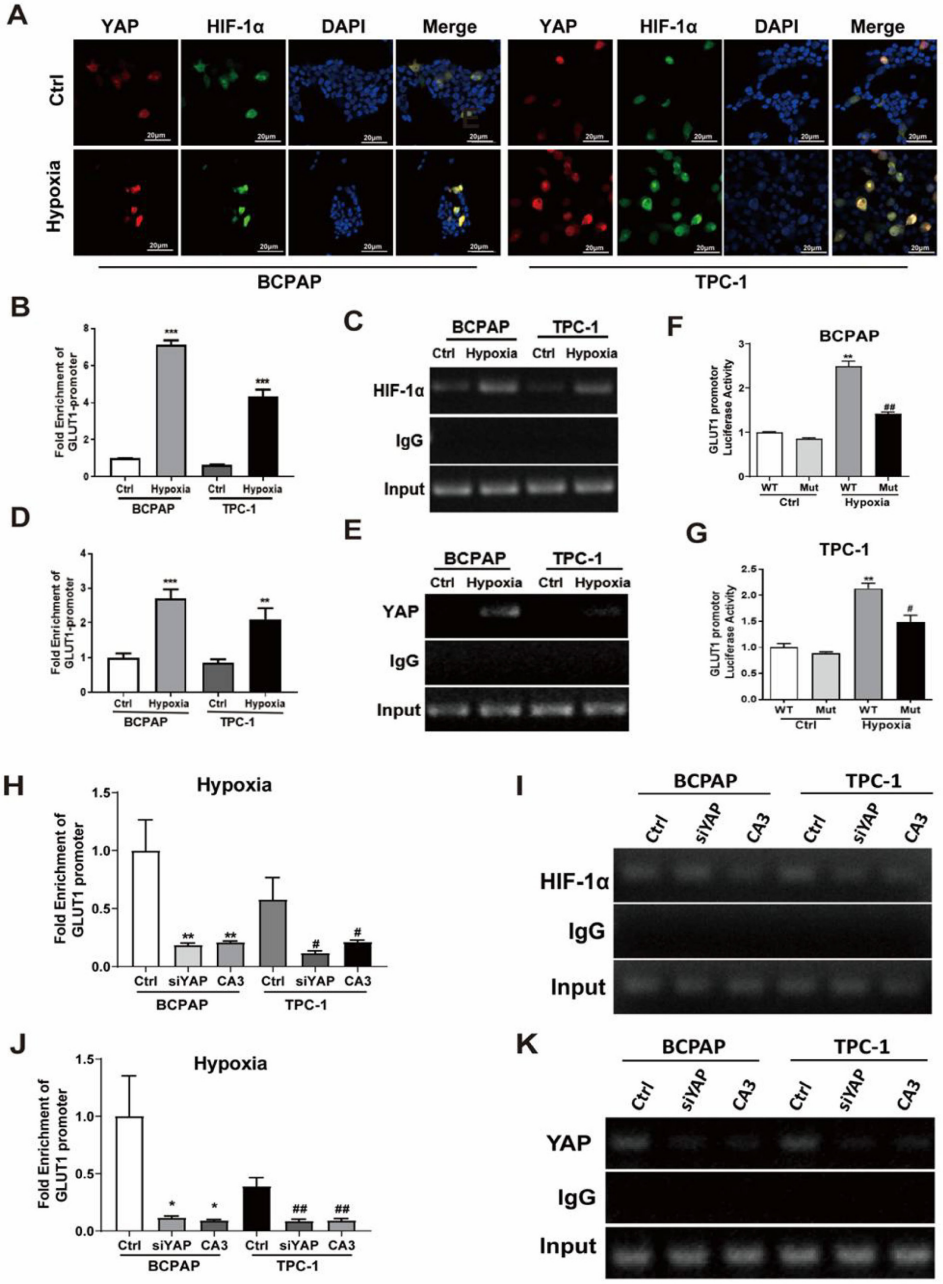
Hypoxia induced YAP nuclear translocation in PTC cells, and the YAP/ HIF-1a complexes bound to GLUT1 gene promoter and directly activated its transcription to accelerate glycolysis under hypoxia. (**A**) Representative immunostaining of HIF-1α and YAP in PTC cells under normoxia (20%O_2_) or hypoxia (1%O_2_) for 24h. (magnification, × 600). (**B-E**) ChIP assay was performed with antibody against HIF-1α, YAP or control IgG in PTC cells under normoxia (20%O_2_) and hypoxia (1%O_2_) for 24 h. Real-time PCR was used to analyze the immunoprecipitated DNA. **p < 0.01 versus IgG, ***p < 0.001versus IgG. (**F-G**) pGL3-WT GLUT1 HRE (wt) or pGL3 mutant GLUT1 HRE (mut) with AGCTACATTA were cotransfected into PTC cells under normoxia (20%O_2_) or hypoxia (1%O_2_) for 24h. Dual-luciferase reporter assay was performed to detect the promoter activity, which was normalized to Renilla luciferase activity. (**H-K**) ChIP assay was performed with antibody against HIF-1α, YAP or control IgG in PTC cells under hypoxia (1%O_2_) for 24h with the inhibition of YAP. Real-time PCR was used to analyze the immunoprecipitated DNA of GLUT1. Data are shown as the mean ± SEM of three independent experiments. *p < 0.05, **p < 0.01, ***p < 0.001, ^#^p < 0.05, ^##^p < 0.01.

The phosphorylation status and localization determines YAP activity and nuclear localization is the most important regulatory mechanism for activating YAP 25. Our previous histological results showed that, in PTC tissues, YAP and HIF1-α showed stronger nuclear staining compared with paracancerous tissues (Fig.1D-E). To determine whether hypoxia activates YAP, we performed western blotting on PTC cell lines. The expression of p-YAP was significantly decreased and HIF1-α was significantly increased under hypoxic conditions. Meanwhile, the protein levels of p-LATS were decreased (Fig. 3C, Supplementary Fig. 1E and F), which was the upstream regulators of YAP in the Hippo pathway. The results suggested that hypoxia might inhibit Hippo signaling, activate YAP and induce YAP nuclear translocation. Further, immunofluorescence staining was performed to examine the cellular localization of HIF1-α and YAP. As shown in Figure 5A, hypoxia strongly promoted the accumulation of HIF1-α and YAP in the nucleus. Thus, we speculated that YAP might complex with HIF-1α in the nucleus under hypoxia in PTC cells.

Poor differentiation in thyroid cancers is associated with increased expression of GLUT1, which is the main glucose transporter that mediated the transport of ^18^F-FDG 3. As shown in Fig. 3A-C, real-time PCR and western blotting both indicated that the expression of GLUT1 was significant increased under hypoxia in the PTC cells, especially in BCPAP. Thus, we proposed a hypothesis that YAP/HIF1-α complexes could bind and activate GLUT1 gene to promote PTC cells glycolysis under hypoxia. In the nucleus, because of the lack of DNA-binding domains, the transcriptional coactivator YAP relies on multiple domains to interact with transcription factors 11. HIF1-α, an oxygen-sensitive transcriptional activator, is generally thought to contribute to the Warburg effect by stimulating many genes that mediate glycolysis, including GLUT1, which has a hypoxia-response elements (HRE) in its promoter. Therefore, we focused on analyzing the HIF1-α and GLUT1 gene sequences and identified a candidate HRE (HIF1-α binding site 5'-GATCGTGCCG-3') in the GLUT1 promoter. To indicate whether YAP/HIF1-α complexes directly regulates the GLUT1 gene at this locus, ChIP assay was performed with antibody against HIF-1α, YAP or control IgG in PTC cells under either hypoxia (1%O_2_) or normoxia (20%O_2_). The results revealed that the pulled DNA by HIF-1α or YAP antibody from PTC cells under hypoxia contained enriched HRE (HIF1-α binding site 5'-GATCGTGCCG-3') sequences, compared with control (Fig. 5B-E). In addition, we have built luciferase reporter plasmids containing either wild or mutant HRE sequences in the GLUT1 promoter and subsequently transfected it into PTC cells. In consistence, the luciferase activity of reporter plasmids with wild‐type of HRE was significantly increased, while plasmids with the mutant‐type of HRE failed to elevate in the PTC cells under hypoxia. To further confirm that YAP was essential for activation of the GLUT1 promoter, with YAP siRNA or CA3 treatment in PTC cells under hypoxia, ChIP assay was performed. As shown in Fig. 5H-K, inhibiting of YAP, the enrichment of GLUT1 with HIF-1α or YAP antibody was significantly decreased compared with control. The above data suggest that hypoxia inhibited the Hippo pathway in PTC cells, activated YAP, and induced YAP translocation to the nucleus. Furthermore, HIF1-α and YAP bound and activated the GLUT1 promoter, enhancing its expression under hypoxia.

### YAP bound and maintained HIF1-α stability in PTC cells under hypoxia

**Figure 6:**
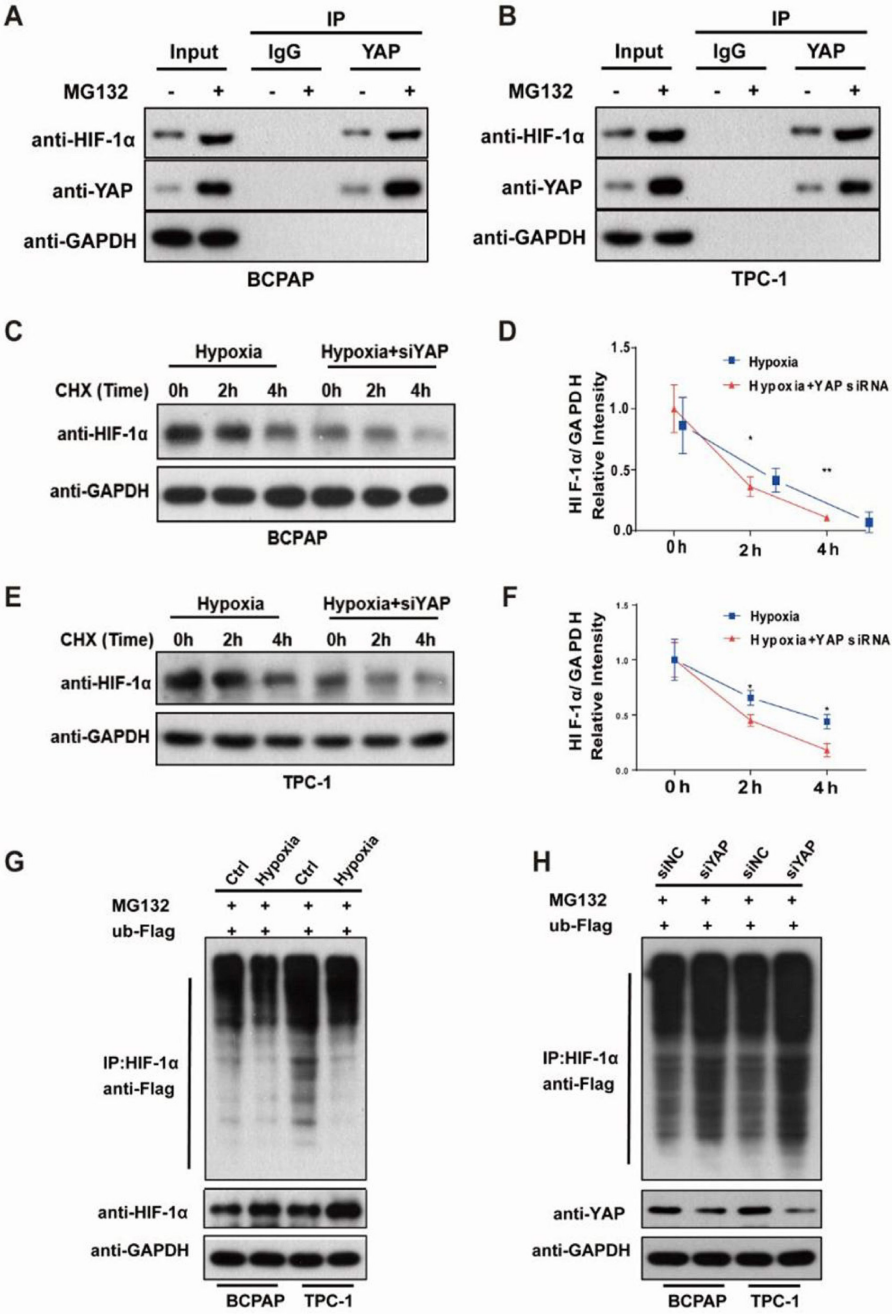
YAP bound and sustained HIF-1α stability in PTC cells under hypoxia. (**A-B**) Co-immunoprecipitation of endogenous YAP in PTC cells under hypoxia (1%O_2_) for 24h in the presence of 10 μM MG132. (**C-F**) Western blot showed the HIF-1α stability in control or siYAP cells treated with 100 μg/ml cycloheximide (CHX) at 0, 2 h and 4 h. (**G**) Degradation of HIF-1α in PTC cells under normoxia (20%O_2_) or hypoxia (1%O_2_) in the presence of 10 μM MG132. (**H**) Degradation of HIF-1α in control or siYAP cells treated with 10 μM MG132.

As we have shown in Figure 5A, hypoxia promoted the accumulation of HIF1-α and YAP in the nucleus and we speculated that YAP might complex with HIF-1α in the nucleus under hypoxia in the PTC cells. Co-immunoprecipitation showed that YAP bound to HIF‑1α as a complex (Fig. 6A-B). Furthermore, with the protease inhibitor MG132 treatment, the binding of YAP to HIF‑1α was increased, indicating that HIF‑1α was degraded through the ubiquitin-proteasome pathway. In addition, we used CHX chase assays to explore the effect of YAP on HIF-1α stability, and found that YAP-silenced PTC cells had significantly shortened HIF-1α half-life compared with the control under hypoxia (Fig. 6C-F). To investigate the degradation of HIF-1α by the ubiquitin-proteasome pathway and confirm the stabilizing effect of YAP on HIF-1α, ubiquitylation assays were performed and the ubiquitylation level of HIF‑1α was detected using an anti-Flag antibody. Indeed, HIF‑1α exhibited ubiquitylation, but its degradation was significantly reduced in PTC cells exposed to hypoxia (Fig. 6G). Conversely, knockdown of YAP markedly increased HIF‑1α ubiquitylation and degradation as revealed by western blotting compared with the control (Fig. 6H). These observations revealed that YAP bound HIF-1α to form a complex and maintained HIF-1α stability under hypoxia.

### Hypoxia inhibited NIS expression, while silencing YAP or CA3 treatment promoted NIS expression in PTC cells under hypoxia

**Figure 7:**
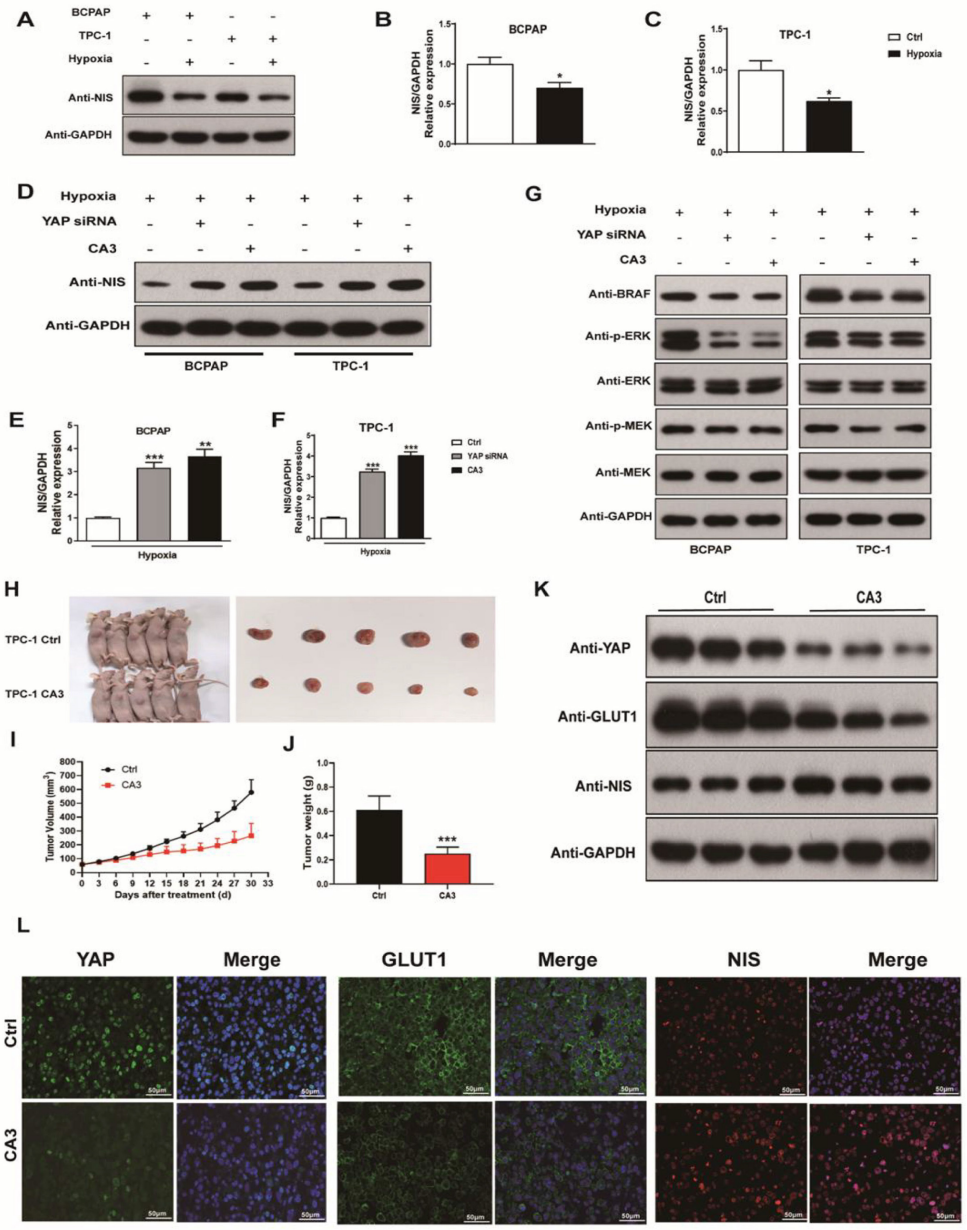
The expression of NIS decreased under hypoxia (1%O_2_) and increased with the inhibition of YAP (silencing YAP or CA3). CA3 treatment inhibited the growth of PTC tumor cells in vivo. (**A**) Western blot showed the expression of NIS protein in BCPAP and TPC-1 cells under normoxia (20%O_2_) or hypoxia (1%O_2_) for 24h, respectively. (**B-C**) Real-time PCR analysis for the expression of NIS gene in BCPAP and TPC-1 cells under normoxia (20%O_2_) or hypoxia (1%O_2_) for 24h, respectively. (**D**) Western blot showed the expression of NIS protein in PTC cells under hypoxia (1%O_2_) for 24h with the inhibition of YAP. (**E-F**) Real-time PCR showed the mRNA expression of NIS gene in PTC cells under hypoxia (1%O_2_) for 24h with the inhibition of YAP. (**G**) Western blot showed the expression of BRAF, p-ERK, ERK, p-MEK, and MEK proteins in PTC cells under hypoxia (1%O_2_) for 24h with the inhibition of YAP. (**H**) Subcutaneous xenografts derived from the control and CA3-treated tumor-bearing mice. (**I**)Tumor growth curve of each group described above. (**J**) Tumor weight of each group described above. (**K**) Western blot showed the expression of YAP, GLUT1, and NIS proteins in control or CA3-treated group. (**L**) Representative immunofluorescence of YAP, GLUT1, and NIS in control or CA3-treated group (magnification, × 400). Data are shown as the mean ± SEM of three independent experiments. *p < 0.05, ***p < 0.001.

To explore the contribution of hypoxia into NIS expression, we incubated PTC cells under hypoxic and normoxic conditions for 24h. The expression of NIS was significantly decreased under hypoxic conditions (Fig. 7A-C). Recent studies implicated RAS genes were transcriptionally regulated by YAP 16. Induction of RAS signaling results in increased MAPK output, which was the most important pathway contributing to NIS downregulation in thyroid cancer. Thus, we examined the effects of YAP on NIS expression. As shown in Figure 7D-F, treatment with YAP siRNA and CA3 under hypoxia both significantly increased NIS expression in PTC cells compared with the control. The results might imply that inhibition of YAP was unable to induce the transcription of oncogenic RAS, leading to decreased MAPK output, which further resulted in increased NIS expression. The subsequent western blotting experiment confirmed our speculation. As shown in Figure 7G, inhibition of YAP under hypoxia in PTC cells, the expression of proteases in the MAPK signaling pathway (BRAF, ERK and MEK) were significantly decreased compared with the control. These results suggested that hypoxia inhibited NIS expression by activating YAP, which promoted the MAPK signaling pathway and ultimately leaded to the reduction of NIS expression.

### CA3 treatment suppressed thyroid tumor growth in vivo

To further confirm the proliferation potential of YAP in PTC progression and its regulatory role in the glucose/iodine metabolism program, TPC-1 cells were subcutaneously injected into nude mice and they were randomly divided into the control and CA3-treated group. As results shown, the size of xenograft tumors from CA3-treated mice was much smaller than the control (Fig. 7H). Furthermore, tumor volumes from CA3-treated mice showed lower growth rates and lighter weight than tumors from the control (Fig. 7I-J). Subsequently, the level of YAP, GLUT1 and NIS were evaluated using immunofluorescent staining. We found that the expression of YAP and GLUT1, which promoted proliferation, were decreased in the CA3-treated group, meanwhile the expression of NIS, which promoted iodine uptake, was increased (Fig. 7G). Collectively, these data demonstrated that HIF-1α/YAP signaling promoted PTC tumor proliferation and regulated glucose/iodine metabolism program in vivo.

## Discussion

In the present study, we found that hypoxia inhibited the Hippo signaling pathway in PTC cells and promoted YAP nuclear localization. YAP activation was critical for accelerating glycolysis and downregulating NIS under hypoxia. Inhibition of YAP had the opposite effects in vitro and tumorigenicity in vivo. Mechanistically, YAP bound to HIF-1α in the nucleus and maintained HIF-1α protein stability. The complex of YAP/HIF-1α bound to the GLUT1 gene and directly activated its transcription to accelerate glycolysis in PTC cells under hypoxia. Meanwhile, YAP might indirectly reduce the expression of NIS by activating the MAPK pathway.

Dedifferentiation of follicle cell-derived thyroid cancer cells is closely related to a loss of iodine uptake; meanwhile, it simultaneously become more eager for nutrients such as glucose 3. Thus, the flip-flop phenomenon (ie, ^131^I negative and ^18^F-FDG positive) has been regarded as a well-described feature of progressive thyroid cancer 26. A high suspicion index must be maintained when this phenomenon occurred, as it associated with the transition from DTC to increasingly aggressive thyroid cancer types, such as poorly DTC and anaplastic thyroid cancer (ATC) 3. Further, this phenomenon was not only observed in different patients, but also in different tumor sites in one patient. Aggressive DTC posed a particularly difficult challenge in thyroid cancer medical treatment regimen. A deeper understanding of the mechanism of glucose/iodine “flip-flop” phenomenon in thyroid cancer may have both prognostic and therapeutic implications.

Historically, the definition of cancer has been described as an abnormal cell proliferation. However, recent evidences proved that cancer is indeed a metabolic disorder. Transformed cancer cells rewire their energy metabolism to meet the demands of increased proliferation and survival in a stressful microenvironment. Increasing evidence supports the notion that reprogramming metabolism is an emerging hallmark of cancer 10. It is well-known that malignant tumors had a high rate of glycolysis and this phenomenon was called Warburg effect, resulting in dramatically increased glucose uptake 8. Recently, the relationship between tumor differentiation and glucose metabolism in thyroid cancer was evaluated. Accumulating evidence supported that high expression of GLUT1 was tightly associated with loss of thyroid tumor differentiation and greater biological invasiveness 5. In addition, within the tumor cell membrane, GLUT1 was the main glucose transporter mediating ^18^F-FDG transport. In our study, immunohistochemical staining data confirmed that the expression of GLUT1 was higher in PTC tissues than in paracarcinoma tissues. Furthermore, real-time PCR and western blotting both indicated that the expression of GLUT1 was significantly increased in the hypoxic PTC cells than in the normoxic. A wealth of evidence from basic science to clinical studies converges that hypoxia was a common phenomenon in various kinds of malignant tumors and played a crucial role in promoting tumor glycolysis, metastasis and invasion. These data consistent with our previous and current research findings 7, 9, 27.

Cancer cells exhibited extensive complexity in reprogramming of glucose metabolism. In addition to the hypoxic tumor microenvironment, dysregulation of certain signaling pathways also contributed to cancer metabolic rewiring. Recently, a connection between glucose metabolism and the Hippo pathway activity has been observed 11, 12, 18. Hippo signaling pathway, as a highly conserved tumor suppressor pathway, mainly included mammalian Ste20-like kinases 1/2 (MST1/2) and large tumor suppressor 1/2 (LATS1/2), YAP and/or its paralog TAZ. YAP was an important transcriptional regulator of the Hippo signaling pathway. It emerged as a growth promoter by modulating cancer cells aggressive behaviors, stem cell diferentiation and chemotherapy resistance, including thyroid cancer 11. SE Lee et al. found that YAP was overexpressed in PTC and ATC. Moreover, in patients with BRAF^V600E^ (+) PTC, nuclear YAP was closely related to the extrathyroidal spread and aggressive features such as local invasion and distant metastasis 28. In other study, Tian Liao et al. reported that YAP was upregulated in human PTC tissues and promoted cancer cell proliferation by activating the ERK/MAPK signaling pathway 29. Consistent with those researches, we also found that YAP was significantly overexpressed in PTC tissues compared to the adjacent normal. What's more, a positive correlation was observed between the expression of YAP and TNM stage in our study. Inhibiting YAP significantly reduced the migration and invasion in vitro and tumorigenicity in vivo. However, the relationship between YAP and PTC glucose metabolic reprograming has been barely investigated.

Recently, novel mechanisms between hypoxia and YAP in cancer glucose metabolism have garnered increasing attention. The phosphorylation status and intracellular localization of YAP determined its activity. YAP nuclear localization was the most important regulatory mechanism for its activation 11. Generally, in the nucleus, YAP mainly relied on multiple domains to interact with transcription factors to form complexes, promoting the expression and activation of downstream target genes 11, 20. Bin Zhu et al. reported that YAP sustained HIF‐1α stability and promoted HIF‐1α's activity on the target gene pyruvate kinase M2 (PKM2), thereby inducing chemoresistance in acute myeloidleukemia under hypoxia 30. Recent study has demonstrated that HMGB1 triggered both YAP and HIF-1α nuclear translocation and enhanced YAP and HIF-1α interaction promoting pancreatic cancer stemness 19. Intriguingly, Zhang et al. shown that YAP interacted directly with HIF-1α in the nucleus and maintained HIF-1α stability, which further promoted hepatocellular carcinoma progression 20. Our findings were in line with those observations that hypoxia induced YAP activation and promoted YAP binding to HIF-1α in the nucleus to stimulate glycolysis by activating GLUT1 in PTC cells. In the present study, we first demonstrated that the expression of HIF1-α and YAP in the PTC specimens were obviously higher than those in paracarcinoma tissues, which were significantly positively correlated with each other (YAP vs HIF1-α, p<0.0001). Then we proved that hypoxia promoted PTC cell glycolysis in vitro, however, YAP inhibition using either siRNA or the CA3 (an inhibitor of YAP) treatment both significantly inhibited PTC cell glycolysis under hypoxia stress. These results indicated that YAP was essential for hypoxia-triggered glycolysis in PTC cells and we speculated that YAP might be involved in promoting PTC cell glycolysis via HIF1-α. Further, the underlying molecular mechanisms involved were investigated.

In mammals, the phosphorylation cascades of Hippo core components inhibited the activation of transcriptional co-activators YAP. LATS was the core protein of the Hippo pathway and an upstream regulator of YAP 11, 12. Our data show that hypoxia reduced p-LATS expression, thereby inhibiting the Hippo pathway and activating YAP. As a transcriptional coactivator of the Hippo signaling pathway, nuclear localization was the most important regulatory mechanism of YAP activation. Our data demonstrated that YAP shown stronger nuclear staining in PTC tissues. Then immunofluorescence staining further verified hypoxia-triggered YAP nuclear translocation in PTC cells, which was consistent with the results of SE Lee et al 28. and Tian Liao et al 29. Furthermore, we observed that in both human PTC tissues and PTC cells, elevated expression of YAP in the nucleus was accompanied by an increase in HIF1-α. Co-immunoprecipitation confirmed that YAP bound to HIF‑1α as a complex. Since inhibition of YAP reduced HIF-1α protein expression, it was possible that YAP was important for HIF-1α stability under hypoxia. CHX chase assays proved this and the findings showed that YAP-silenced PTC cells had significantly shortened HIF-1α half-life compared with the control under hypoxia. The ubiquitination assay further verified the stabilization of YAP on HIF-1α. Interestingly, it was reported that YAP initiated the transcription by cooperating with other transcription regulators to control many genes related to glycolysis. Meanwhile, HIF1-α, as an oxygen-sensitive transcriptional activator, was suggested to stimulating a number of genes that mediated glycolysis. Thus, we identified a candidate HRE (HIF1-α binding site 5'-GATCGTGCCG-3') in the GLUT1 promoter and the complex of YAP/HIF1-α directly activated its transcription. Taken together, hypoxic stress in the PTC cells promoted YAP binding to HIF-1α in the nucleus and maintained HIF-1α protein stability to promote glycolysis by activating GLUT1.

In the present study, we found that hypoxic stress not only promoted the expression of GLUT1, but also simultaneously inhibited the expression of NIS. Real-time PCR and western blotting both indicated that the expression of NIS was significant decreased under hypoxia in the PTC cells. Castillo-Rivera et al. have provided evidence that hypoxia impaired NIS expression at the plasma membrane and iodide uptake within tumors, which findings were consistent with our results 31. To demonstrate whether YAP was involved in the regulation of NIS expression, real-time PCR and western blotting were performed in PTC cells under hypoxia. The data showed that NIS expression was significantly increased in YAP-inhibited PTC cells under hypoxia compared to control. These results indicated that YAP was essential for hypoxia-triggered NIS downregulation in PTC cells. As is known, genetic aberrations (such as RAS and BRAF) are prominently responsible for the onset, progression, and dedifferentiation of PTC, mainly through the activation of MAPK signaling pathways. Eventually, these alterations result in a lack of NIS and disabling of RAI uptake, leading to the development of RAIR-PTC 32. It has been reported that YAP induced the proliferation of PTC cells in vitro and in vivo by stimulating MAPK signaling 33. Furthemore, recent studies implicated RAS genes are transcriptionally regulated by YAP 16. Induction of RAS signaling results in increased MAPK output, which is the most important pathway contributing to NIS downregulation in thyroid cancer. Therefore, we speculated that YAP might regulated the expression of NIS by MAPK signaling in PTC cells under hypoxia. The subsequent western blotting experiment confirmed it and inhibition of YAP under hypoxia significantly decreased the expression of proteases in the MAPK signaling pathway (BRAF, ERK and MEK) compared with the control. These results suggested that activated HIF-1α/YAP signaling promoted the MAPK signaling pathway, which might indirectly leaded to the reduction of NIS.

In the present study, by exploring the underlying mechanism of glucose/iodine “flip-flop” phenomenon, we proposed a novel theory to PTC progression from a metabolic perspective. Nonetheless, there are some limitations to this study. First, our data showed that hypoxia decreased p-LATS expression to inhibit the Hippo pathway and activate YAP, but the specific mechanism remained to be studied in PTC. Second, we have provided evidence that HIF-1α/YAP signaling might indirectly reduce NIS expression by activating the MAPK pathway, promoting cell proliferation in PTC. However, further investigation of the underlying mechanism is still needed to determine how HIF-1α/YAP signaling triggers the MAPK pathway. These are what we need to explore in the future.

## Conclusions

In summary, our study demonstrated that YAP expression was higher in PTC tissues and correlated positively with HIF-1α, GLUT1 and TNM stage. hypoxia-induced YAP activation was essential for accelerating PTC cell glycolysis and reducing NIS expression. Withdrawal of YAP had the opposite effects in vitro and tumorigenicity in vivo. YAP activation interacted directly with HIF-1α in the nucleus and sustained HIF-1α stability to activate GLUT1 transcription. Meanwhile, HIF-1α/YAP signaling might indirectly reduce the expression of NIS by activating the MAPK pathway. Thus, by exploring the underlying mechanism of iodine/glucose “flip-flop” phenomenon, we propose a novel theory to promote the progression of PTC. These preclinical findings suggest that the inhibition of HIF-1α/YAP signaling, alone or in combination with other potential markers, may effectively combat aggressive PTC. In the near future, it will most likely introduce altered metabolic pathways as therapeutic targets of thyroid cancers.

## Supplementary Material

Supplementary figures, table and file.

## Figures and Tables

**Figure 8:**
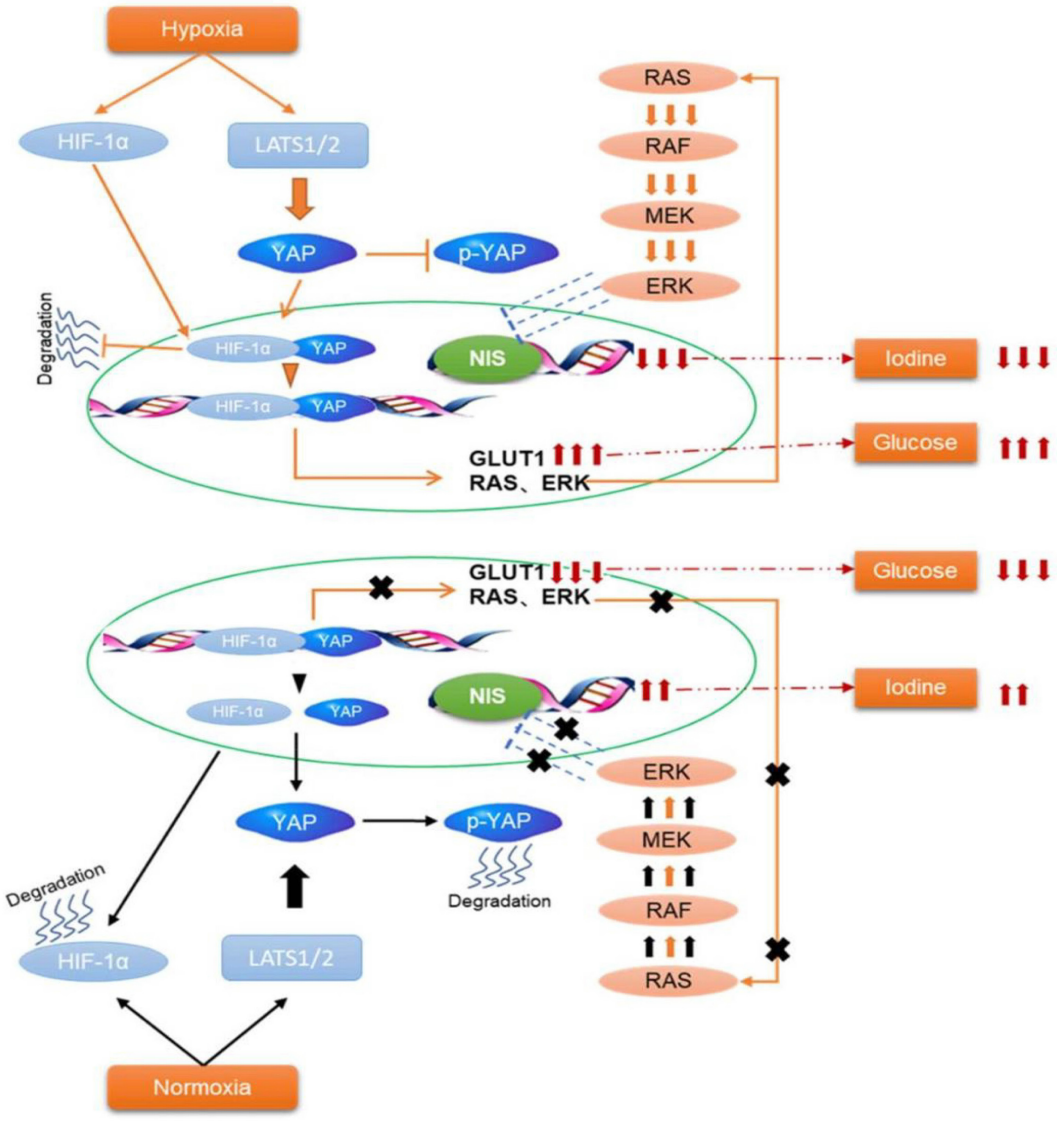
Schematic representation of HIF-1α/YAP signaling mediating glucose/iodine metabolism program in papillary thyroid carcinoma. The HIF-1α/YAP complexes bound to GLUT1 gene promoter and directly activated its transcription to accelerate glycolysis under hypoxia. Meanwhile, it indirectly activates the MAPK signaling pathway, resulting in a decrease in NIS and iodine uptake.

## Abbreviations

T: tumor
N: normal
HIF-1a: hypoxia inducible factor-1a
YAP: Yes-associated protein
GLUT1: glucose transporters 1
PTC: papillary thyroid carcinoma
IHC: immunohistochemistry
TNM: tumor-node-metastasis
ECAR: extracellular acidification rate
GLU: glucose
OLI: oligomycin
2-DG: 2-deoxyglucose
HK2: hexokinase 2
PKM2: Pyruvate kinase M2
LDHA: Lactate dehydrogenase A
PGK1: Phosphoglycerate kinase 1
LATS: Large tumor suppressor kinase
CHX: Cycloheximide
NIS: sodium iodide symporter
MAPK: mitogen-activated protein kinase
