# Supplementary material for: HIF-1α/YAP Signaling Rewrites Glucose/Iodine Metabolism Program to Promote Papillary Thyroid Cancer Progression
Source: Int J Biol Sci. 2023 Jan 1;19(1):225–41. doi: 10.7150/ijbs.75459 (PMC9760428; doi:10.7150/ijbs.75459)
Supplement: Supplementary file 1 — Supplementary figures, table and file. [file ijbsv19p0225s1.pdf]

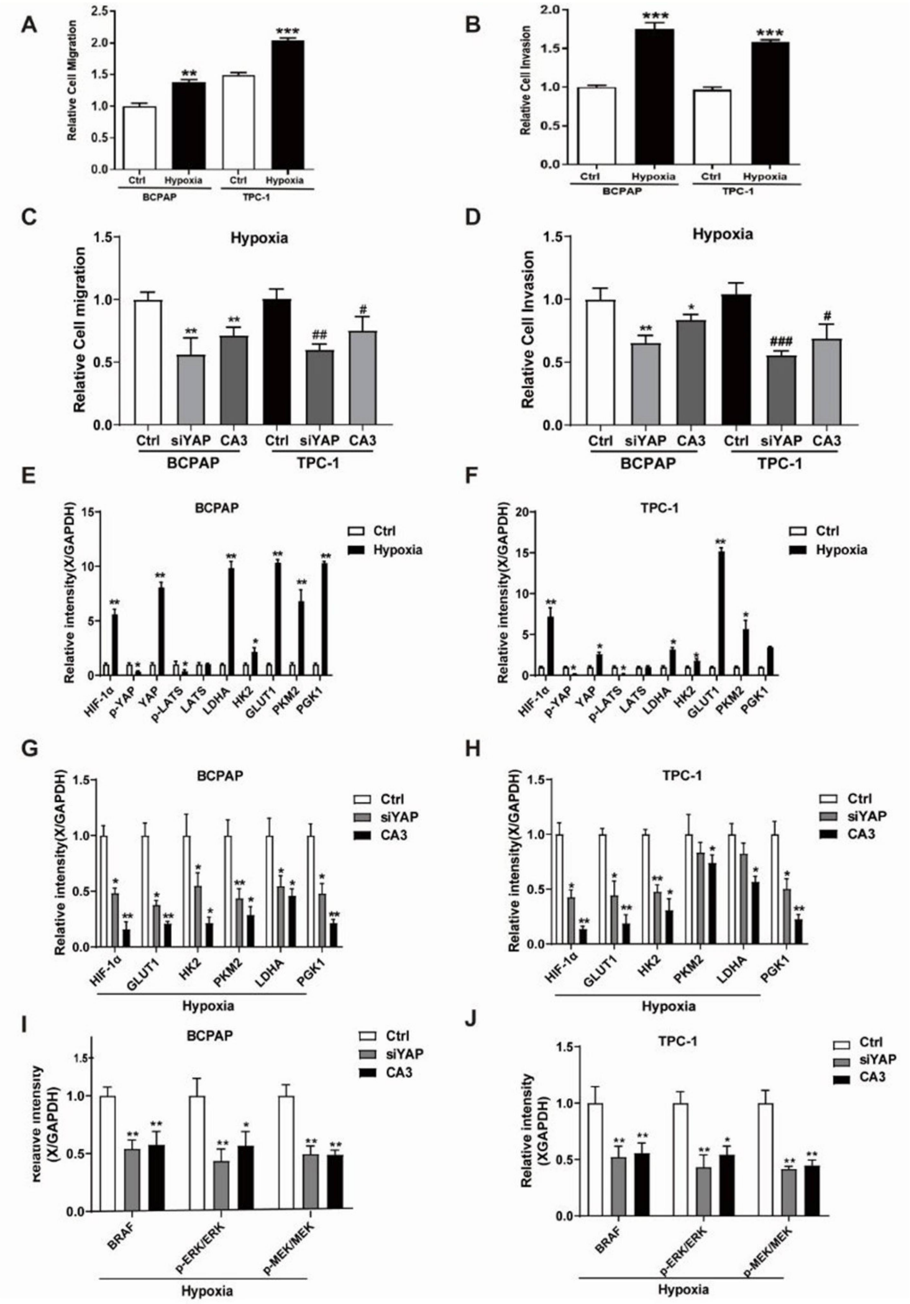

Figure S1.

Semi-quantitative histogram of Western blot proteins. (**A-B**) Migration and invasion assays in BCPAP and TPC-1 cells under normoxia (20% O_2) or hypoxia (1% O_2) for 24h. (**C-D**) Western blot showed the expression of HIF-1a, YAP, LATS, LDHA, HK2, GLUT1, PKM2, and PGK1 proteins in BCPAP and TPC-1 cells under normoxia (20%O_2) or hypoxia (1%O_2) for 24h, respectively. (**E-F**) Migration and invasion assays in BCPAP and TPC-1 cells under hypoxia (1% O_2) for 24h after transfected with YAP siRNA or the use of the inhibitor CA3. (**G-H**) Western blot showed the expression of HIF-1a, GLUT1, HK2, PKM2, LDHA and PGK1 proteins in BCPAP

and TPC-1 cells under hypoxia (1%O_2) for 24h with the inhibition of YAP. (**I-J**) Western blot showed the expression of BRAF, p-ERK/ERK and p-MEK/MEK proteins in PTC cells under hypoxia (1%O_2) for 24h with the inhibition of YAP.

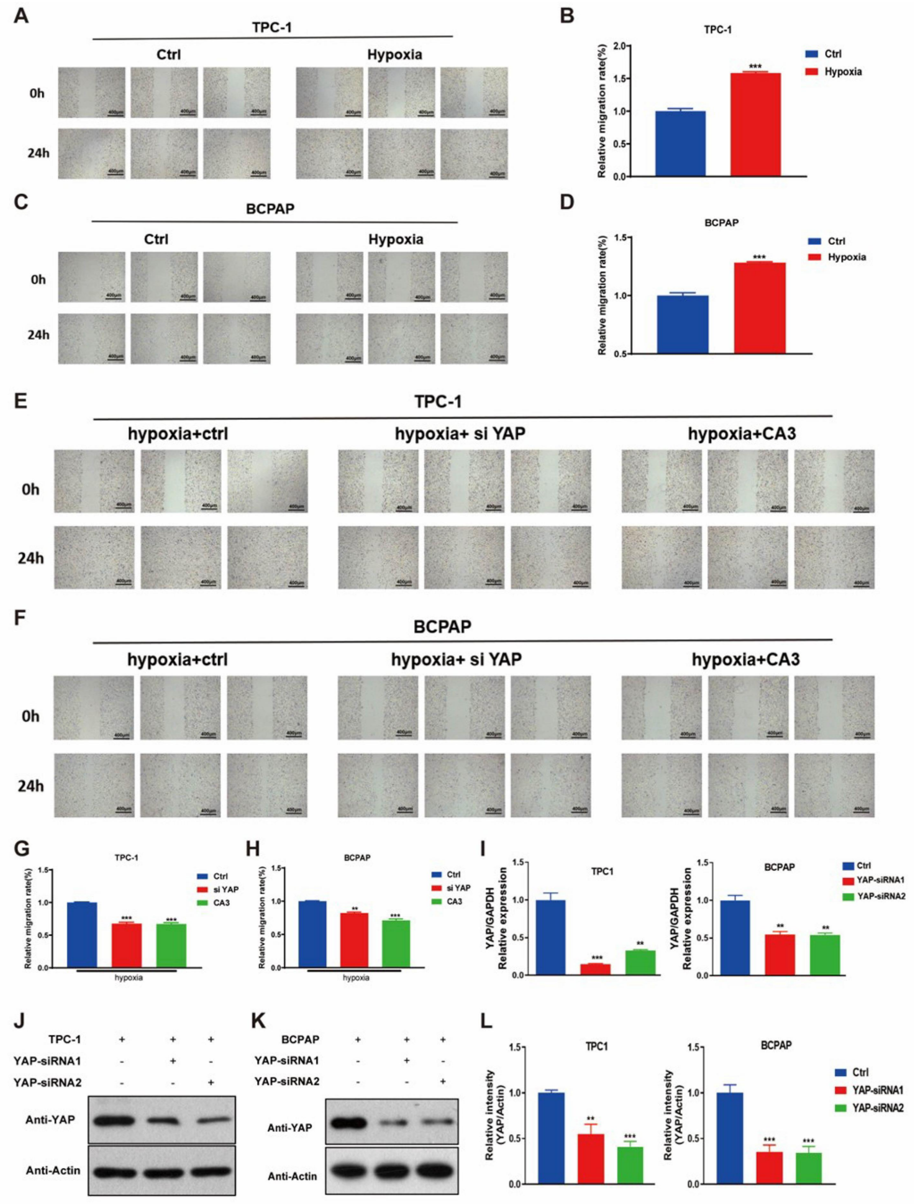

Figure S2.

(A-D) Migration and invasion assays in BCPAP and TPC-1 cells under normoxia (20% O_2) or hypoxia (1% O_2) for 24h (wound healing assays). Data are shown as the mean ± SEM of three independent experiments. **(E-H)** Migration and invasion assays in BCPAP and TPC-1 cells under hypoxia (1% O2) for 24h after transfected with YAP siRNA or the use of the inhibitor CA3. (wound healing assays). **(I-L)** Real-time PCR and western blot were used to examine knockdown

efficiency of YAP in BCPAP and TPC-1 cells transfected with YAP siRNA. **$p < 0.01$, ***$p < 0.001$.

Supplementary Table 1.

Information about the western blotting antibodies in the study

Antibody Name	Source	Dilution Ratio	Company	Item Number
GLUT1	rabbit	1:2000	abcam	ab15730
HIF-1α	mouse	1:1000	cst	#79233T
Sodium Iodide Symporter (NIS)	rabbit	1:1000	abcam	ab83816
p-YAP	rabbit	1:1000	cst	#13008
YAP	rabbit	1:1000	cst	#14074T
p-LATS	rabbit	1:1000	cst	9157S
LATS	rabbit	1:1000	abcam	ab243656
LDHA	rabbit	1:1000	cst	3582
HK2	rabbit	1:1000	cst	#2867
PKM2	rabbit	1:1000	abcam	ab137852
PGK1	rabbit	1:1000	abcam	ab199438
Flag	mouse	1:1000	proteintech	66008-2-ig
Histone H3	rabbit	1:1000	abcam	ab5103
GAPDH	mouse	1:20000	cst	#5174
Anti-mouse IgG(HRP)		1:5000	cst	#7076
Anti-rabbit IgG(HRP)		1:5000	cst	#7074

Supplementary file 1.

Real-time PCR primers

YAP	Forward: 5′-TAGCCCTGCGTAGCCAGTTA-3′	
	Reverse: 5′-TCATGCTTAGTCCACTGTCTGT-3′	
HIF-1α	Forward: 5′-ATCCATGTGACCATGAGGAAATG-3′	
	Reverse: 5′-TCGGCTAGTTAGGGTACACTTC-3′	
GLUT1	Forward: 5′-TCTGGCATCAACGCTGTCTTC-3′	
	Reverse: 5′-CGATACCGGAGCCAATGGT-3′	
HK2	Forward: 5′-TTGACCAGGAGATTGACATGGG-3′	
	Reverse: 5′-CAACCGCATCAGGACCTCA-3′	
PKM2	Forward: 5′-ATAACGCCTACATGGAAAAGTGT-3′	
	Reverse: 5′-TAAGCCCATCATCCACGTAGA-3′	
LDHA	Forward: 5′-ATGGCAACTCTAAAGGATCAGC-3′	
	Reverse: 5′-CCAACCCCAACAACTGTAATCT-3′	
PGK1	Forward: 5′-GACCTAATGTCCAAAGCTGAGAA-3′	
	Reverse: 5′-CAGCAGGTATGCCAGAAGCC-3′	
NIS	Forward: 5′-CCTATCGCTATGGCCTCAAGT-3′	
	Reverse: 5′-CGTGGCTACAATGTACTGCAAA-3′	
GAPDH	Forward: 5′-GGAGCGAGATCCCTCCAAAAT-3′	
	Reverse: 5′-GGCTGTTGTCATACTTCTCATGG-3′	
GLUT1-WT	Forward: 5′-CTTACGCGTGCTAGCCTGGTCGGCTCAGCCTCCCG-3′	
	Reverse: 5′-CGGAATGCCAAGCTTCTGTCCTGGGCGGCGCTCTG-3′	
GLUT1-mut	Forward: 5′-CCGAAGCTCATTACTGCACTCCAGCCTGG-3′	
	Reverse: 5′-GCAGTAATGAGCTTCGGCTCACTGTAACC-3′	
